# LPAL2 Suppresses Tumor Growth and Metastasis of Hepatocellular Carcinoma by Modulating MMP9 Expression

**DOI:** 10.3390/cells11162610

**Published:** 2022-08-22

**Authors:** Yang-Hsiang Lin, Yu-Chin Liu, Cheng-Yi Chen, Hsiang-Cheng Chi, Meng-Han Wu, Po-Shuan Huang, Cheng-Chih Chang, Tzu-Kang Lin, Chau-Ting Yeh, Kwang-Huei Lin

**Affiliations:** 1 Liver Research Center, Chang Gung Memorial Hospital, Linkou, Taoyuan 333, Taiwan; 2 Molecular Medicine Research Center, Chang Gung University, Taoyuan 244, Taiwan; 3 Graduate Institute of Biomedical Sciences, College of Medicine, Chang Gung University, Taoyuan 33302, Taiwan; 4 Department of Biochemistry, College of Medicine, Chang Gung University, Taoyuan 333, Taiwan; 5 Department of Cell Biology and Anatomy, College of Medicine, National Cheng Kung University, Tainan 701, Taiwan; 6 Graduate Institute of Integrated Medicine, China Medical University, Taichung 40447, Taiwan; 7 Chinese Medicine Research Center, China Medical University, Taichung 406040, Taiwan; 8 Department of General Surgery, Chang Gung Memorial Hospital at Chia-yi, Chia-yi 613, Taiwan; 9 Neurosurgery, School of Medicine, College of Medicine, Fu Jen Catholic University, New Taipei City 24205, Taiwan; 10 Neurosurgery, Department of Surgery, Fu Jen Catholic University Hospital, New Taipei City 24352, Taiwan; 11 Research Center for Chinese Herbal Medicine, College of Human Ecology, Chang Gung University of Science and Technology, Taoyuan 33303, Taiwan

**Keywords:** hepatocellular carcinoma, LPAL2, metastasis, MMP9, cancer stem cell

## Abstract

Tumor metastasis is a complex process modulated by both intrinsic and extrinsic factors that ultimately result in poorer patient outcomes, including diminished survival. Pseudogene-derived long non-coding RNAs (lncRNA) play important roles in cancer progression. In the current study, we found that the pseudogene-derived lncRNA LPAL2 is downregulated in hepatocellular carcinoma (HCC) tissues, and further showed that elevated LPAL2 expression is positively correlated with survival outcome. The knockdown of LPAL2 in hepatoma cells induced tumor formation, migration, invasion, sphere formation, and drug resistance. Metalloproteinase 9 (*MMP9*) was identified as an LPAL2-regulated target gene, consistent with clinical findings that LPAL2 expression is significantly associated with MMP9 expression. Furthermore, patients with a higher expression of LPAL2 and lower expression of MMP9 (LPAL2-high/MMP9-low) had a higher survival rate than those with other combinations. Collectively, our findings establish LPAL2 as a novel tumor suppressor in HCC, and suggest targeting LPAL2 and MMP9 as a therapeutic approach for the treatment of HCC.

## 1. Introduction

Hepatocellular carcinoma (HCC) is among the leading causes of cancer-related deaths [1]. Tumor metastasis, a complex process involving crosstalk among multiple signaling pathways that can be modulated by both intrinsic and extrinsic stimulation [2], is the major cause of poorer survival outcomes among HCC patients. Accordingly, solving the puzzle of metastasis is important for prolonging patient survival rates.

With the advent of RNA-sequencing technology, pseudogenes and non-coding genes, once considered “junk” DNA [3], have been found to function as regulators of cellular functions that act via several mechanisms, including the regulation of gene transcription, regulation of protein stability, and modulation of signal transduction. Non-coding genes can be transcribed to RNA molecules, but are not further translated to a functional protein. These non-coding RNAs (ncRNAs), defined based on a cutoff of 200 nucleotides, are divided into two classes based on length: small ncRNAs and long ncRNAs (lncRNAs) [4]. Pseudogene-derived lncRNAs have also recently been demonstrated to perform unique functions in cells [5]. Kong et al. [6] demonstrated that the pseudogene-derived lncRNA protein disulfide isomerase family A member 3 pseudogene 1 (PDIA3P1) is highly expressed in HCC tissues, and showed that the knockdown of PDIA3P1 repressed the cell growth, migration, and invasion of HCC cell lines through the modulation of the p53 pathway. Other evidence revealed that the expression of the pseudogene-derived lncRNA ubiquitin conjugating enzyme E2 C pseudogene 3 (UBE2CP3) was higher in HCC tissues than in adjacent normal tissues [7]. The ectopic expression of UBE2CP3 was shown to induce the migration and invasion of HCC cell lines, as well as positively regulate the expression of the epithelial to mesenchymal transition (EMT)-related genes Snail and N-cadherin. Accordingly, the pseudogene-derived lncRNA plays a pivotal role in regulating HCC progression.

A previous study identified that a single-nucleotide polymorphism, rs3088442, in solute carrier family 22 member 3-lipoprotein(a)-like 2, pseudogene (LPAL2)-LPA cluster was significantly correlated with the plasma concentration of lipoprotein(a) and coronary artery disease [8]. LPAL2 was identified as a pseudogene-derived lncRNA, but its functional role in cancer progression is limited. Recently, the LPAL2, acting through a signaling cascade involving the microRNA miR-1287-5p and epidermal growth factor receptor (LPAL2/miR-1287-5p/EGFR), was found to be associated with thyroid eye disease [9]. However, the underlying mechanisms of LPAL2 actions, and their clinical significance in HCC, remain unclear. In this study, we found that LPAL2 acts as a tumor suppressor lncRNA in HCC cell lines. In vitro and in vivo models support the conclusion that LPAL2 modulates tumor growth, metastasis and stemness phenotypes of HCC cell lines, and microarray profiling analysis and validation results suggest that LPAL2 regulates metalloproteinase-9 (MMP9) at the transcriptional level. Consistent with this, we found that a higher expression of LPAL2 and lower expression of MMP9 were associated with better prognosis among HCC patients.

## 2. Materials and Methods

### 2.1. Cell Culture and Reagents

The hepatoma cell lines used in this study were maintained in Dulbecco’s modified Eagle’s medium (DMEM), containing 10% (*v*/*v*) fetal bovine serum (FBS). All cell lines were grown at 37 °C in a humidified atmosphere of 95% air and 5% CO_2_. The identity of cell lines was authenticated using the StemElite ID System (Promega Corporation, Madison, WI, USA), and verified by uploading results to the American Type Culture Collection (ATCC) website. Functional assays in this study used cells at passage numbers 5–20. Doxorubicin was purchased from Sigma-Aldrich (St. Louis, MO, USA).

### 2.2. HCC Specimens

The HCC specimens were purchased from Taiwan Liver Cancer Network (TLCN). The protocol was approved by the Medical Ethics and Human Clinical Trial Committee at Chang-Gung Memorial Hospital (IRB no: 202002135B0).

### 2.3. Quantitative Reverse Transcription-Polymerase Chain Reaction (qRT-PCR)

For quantification of LPAL2, MMP9 and 18s rRNA, total RNA was extracted from cells using a TOOLSmart RNA Extractor (BIOTOOLS Co., Ltd., Taipei, Taiwan; DPT-BD24) and converted into cDNA using ToolScript MMLV RTase (BIOTOOLS; TGERA04). qRT-PCR was performed using specific forward and reverse primers and TOOLS SuperFast SYBR qPCR Reagent (with ROX dye) (BIOTOOLS; FPT-BB07). Standard qRT-PCR cycling conditions were 95 °C for 10 min followed by 40 cycles of 95 °C for 15 s and 60 °C for 1 min, with a final dissociation step. Primer sequences are listed in Supplementary Table S1.

### 2.4. Immunoblot Analysis

Immunoblot analyses were performed as described previously [10]. Antibodies specific for MMP9 (Santa Cruz Biotechnology, Inc., Dallas, TX, USA; sc-6841), Bid (Cell Signaling Technology, Boston, MA, USA, #2002), cleaved-PARP (Cell Signaling, #5625), cleaved-caspase 3 (Cell Signaling, #9664), and GAPDH (Santa Cruz; sc-32233) were used. Immunoreactive proteins were detected by chemiluminescence using X-ray films.

### 2.5. Establishment of Cell Lines Stably Overexpressing LPAL2-Targeting shRNAs

Small hairpin (inhibitory) RNAs (shRNAs) specific for LPAL2 (shLPAL2#1 and shLPAL2#2) were designed and cloned into the pLKO vector according to guidelines of the RNAi core laboratory. Each shRNA plasmid and virus packaging-related plasmids, including pCMV-ΔR8.91 and pMD.G, were co-transfected into 293T cells, and virus was collected 48 and 72 h after transfection. Stable LPAL2-knockdown clones were selected by incubating the pool of transfected hepatoma cell lines with medium containing puromycin (1 μg/mL). LPAL2-knockdown efficiency was further measured using qRT-PCR. The shRNA sequences used are listed in Supplementary Table S1.

### 2.6. In Vitro Migration and Invasion Assays

Transwell migration and invasive assays were performed using Boyden chambers (Becton-Dickinson, Franklin Lakes, NJ, USA; #3422). The number of cells seeded on non-Matrigel-coated (migration assay) or Matrigel-coated (invasion assay) upper chambers was adjusted to 6 × 10^5^ and 6 × 10^8^ cells/mL, respectively. After incubation for 16–24 h at 37 °C, cells migrating or invading from the upper to the lower chamber were determined by crystal violet staining and quantified using Image J software (NIH, MD, USA, version 1.41).

### 2.7. Cell Viability and Growth Assays

Cells (5 × 10^3/^ well) were seeded onto 48-well plates. At different time-points, as indicated in the corresponding figure legends, cell proliferation rate and cell viability were measured using MTT [3-(4,5-dimethylthiazol-2-yl)-2,5-diphenyltetrazolium bromide] assays (Thermo Fisher Scientific, Waltham, MA USA; M6494). Absorbance and background were determined at 570 and 650 nm, respectively, using a Titertek Multiskan Plus MK1-ELISA reader (Labsystems and Life Science International Ltd., Haverhill, UK).

### 2.8. Cell Sorting and Flow Cytometry

Sorting of CD133-positive and -negative hepatoma cell lines by flow cytometry was performed using phycoerythrin (PE)-conjugated mouse monoclonal anti-human CD133/1 (Miltenyi Biotec, Bergisch Gladbach, Germany). Isotype control mouse IgG1k-PE (eBioscience, San Diego, CA, USA) was used as a negative control.

### 2.9. Sphere-Formation Assay

LPAL2-knockdown and control cells (1 × 10^3^) were plated onto ultralow-attachment 6-well plates (Corning Inc., Corning, NY, USA) in DMEM/F12 medium containing 20 ng/mL basic fibroblast growth factor, 20 ng/mL epidermal growth factor, and B27. After 3 wk, the number and size of spheres formed were determined using Image J software (Version 1.41).

### 2.10. Animal Models

The effects of LPAL2 on tumor formation were tested by assaying tumor growth in 4-wk-old male BALB/c nude mice subcutaneously injected in the right flank with Huh7-control, Huh7-shLPAL2#1 or Huh7-shLPAL2#2 cells. Tumor volume (mm^3^) was determined every 4 d according to the formula (W^2^ × L)/2, where W is the smallest tumor dimension and L is the longest dimension. Mice from each group were sacrificed 30 d after injection. All assays were performed in a blinded and randomized manner and conformed to guidelines of the United States National Institutes of Health and the Chang Gang Institutional Animal Care and Use Committee Guide for the Care and Use of Laboratory animals (CGU109-141).

### 2.11. Integration of the Protein-Protein Interaction (PPI) Network

The search tool for the retrieval of interacting genes (STRING version 11, https://string-db.org/ (accessed on 1 June 2021)) was applied for the investigation of potential differentially expressed gene (DEG) interactions. The score > 0.4 of protein–protein interaction was considered to map PPI network.

### 2.12. Statistical Analysis

All results are shown as means ± standard deviation (SD) of three independent experiments. Statistical calculations were performed with SPSS version 20 (SPSS Inc., Chicago, IL, USA) using the Mann–Whitney test for comparison of two groups and one-way analysis of variance (ANOVA), followed by Tukey post hoc test for comparisons of two or more groups. Clinical outcomes (overall survival and recurrence-free survival) were measured based on Kaplan–Meier survival curves using the log-rank test. *p* values < 0.05 were considered significant; individual *p*-values (* *p* < 0.01, ** *p* < 0.05) are indicated in figure legends.

## 3. Results

### 3.1. Elevated Expression of LPAL2 in HCC Is Positively Correlated with Clinical Outcome

**Figure 1:**
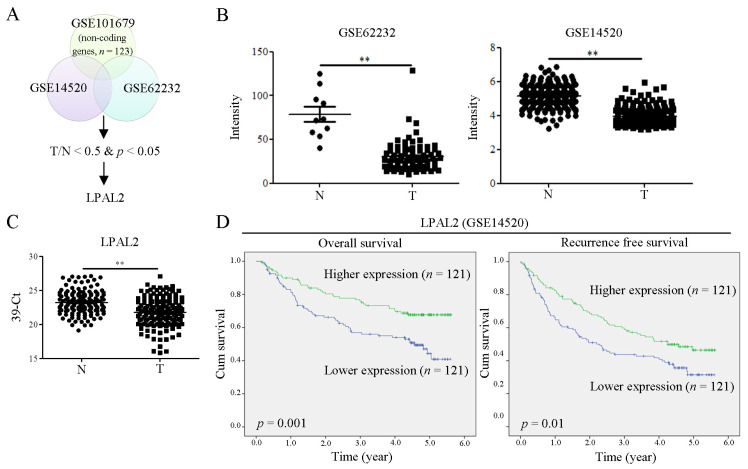
LPAP2 expression is downregulated in HCC tissues and is positively correlated with survival outcomes. (**A**) Differential expression of lncRNAs in tumor (T) and normal (N) tissue in HCC specimens (T/N < 0.5) was analyzed by microarray profiling analysis, which was uploaded to NCBI’s GEO dataset (GES101679). Identified targets were subsequently correlated with HCC tumors using online-available GSE14520 and GSE62232 datasets, an analysis that showed that LPAL2 expression was low in HCC tumors. (**B**) Intensity of LPAL2 in GSE62232 and GSE14520 datasets is shown. **, *p* < 0.01. (**C**) LPAL2 expression levels in our cohort of HCC specimens (*n* = 157), determined by qRT-PCR. 18s rRNA was used as a loading control. **, *p* < 0.01. (**D**) Effect of LPAL2 expression on survival outcomes was determined by Kaplan–Meier survival curve analysis using a log-rank test.

To determine differentially expressed non-coding genes in HCC, we performed a microarray analysis. The expression levels of target genes with HCC were further correlated using the online-available datasets GSE62232 and GSE14520 (Figure 1A). The initial focus was on putative lncRNAs downregulated in HCC. Among these, we found that LPAL2 lncRNA was significantly downregulated in HCC specimens in both datasets (Figure 1B). A qRT-PCR analysis, used to verify microarray profiling, confirmed that LPAL2 expression was lower in HCC tissues compared with adjacent normal tissues (Figure 1C). Notably, a higher expression of LPAL2 was positively correlated with overall and recurrence-free survival (Figure 1D). However, the negative association among LPAL2 expression, tumor size, and tumor grade was not observed in Table 1. This may be due to the small patient sample size. Taken together, these findings suggest that LPAL2 is clinically relevant in HCC.

### 3.2. Knockdown of LPAL2 Enhances Tumor Growth, Migration, and Invasion

**Figure 2:**
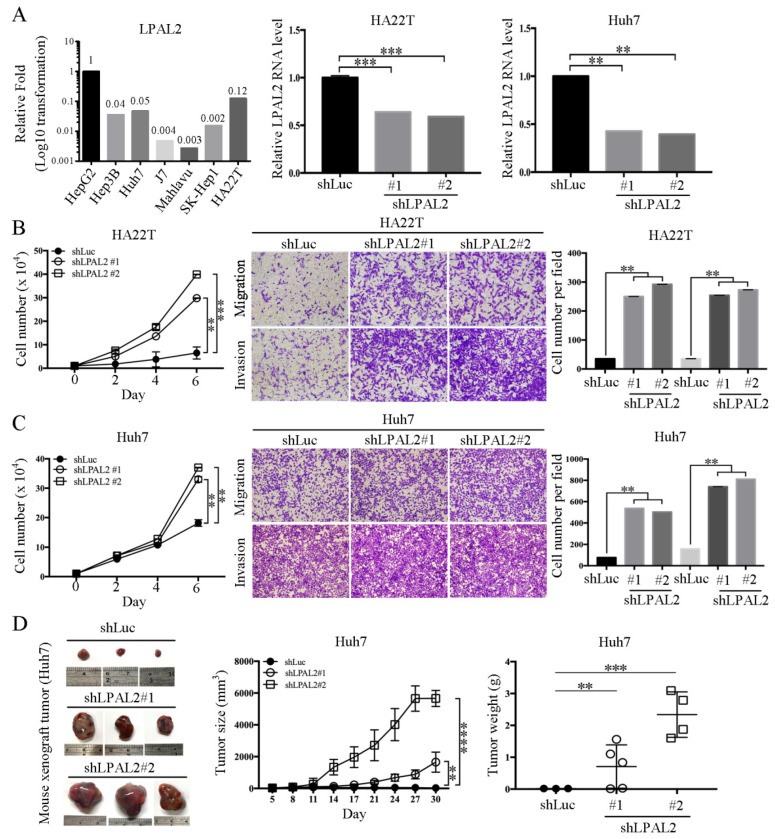
Knockdown of LPAL2 accelerates tumor growth, migration, and invasion. (**A**) Left panel: Endogenous expression level of LPAL2 in hepatoma cell lines (HepG2, Hep3B, Huh7, J7, Mahlavu, SK-Hep1 and HA22T cells) was determined using qRT-PCR. 18s rRNA was used as a loading control. Expression levels of LPAL2 are presented as log10-transformed values. In addition, the average fold-change value in each cell line was also shown. Middle and right panels: Knockdown efficiency of LPAL2 in HA22T and Huh7 cell lines was tested by qRT-PCR. 18s rRNA served as a loading control. **, *p* < 0.01; ***, *p* < 0.001. (**B**,**C**) Cell proliferation, migration, and invasion assays were performed using LPAL2-depleted HA22T and Huh7 cell lines. The migratory or invasive cells were detected by crystal violet staining. **, *p* < 0.01; ***, *p* < 0.001. (**D**) Tumor growth rate and tumor weight were determined in nude mice injected with Huh7-shLuc (control, *n* = 3), Huh7-shLPAL2#1 (*n* = 5) or Huh7-shLPAL2#2 (*n* = 4) cells, respectively.**, *p* < 0.01; ***, *p* < 0.001; ****, *p* < 0.0001;

To date, the exact functional roles of LPAL2 in hepatoma remain unclear. To address this question, we first assessed endogenous LPAL2 expression in hepatoma cell lines. We found that LPAL2 expression levels were higher in HepG2, Hep3B, Huh7 and HA22T cell lines, and lower in J7, Mahlavu and SK-Hep1 (Figure 2A, left panel). As HepG2 is a non-tumorigenic cell line, we established stable lines of HA22T and Huh7 hepatoma cells expressing shRNA against LPAL2, and confirmed the knockdown efficiency of LPAL2 in these cell lines using qRT-PCR (Figure 2A, middle and right panels). We found that the knockdown of LPAL2 in HA22T and Huh7 cell lines accelerated cell growth, migration, and invasion (Figure 2B,C). To verify that the phenotype of LPAL2 in vivo was similar to that in vitro, we subcutaneously injected control (shLuc, *n* = 3) or LPAL2-knockdown (shLPAL2#1, *n* = 5 and shLPAL2#2, *n* = 4) cell lines into nude mice. The depletion of LPAL2 induced an increase in tumor growth and tumor weight (Figure 2D). Taken together, these findings support the conclusion that LPAL2 is a tumor suppressor in HCC.

### 3.3. MMP9 Is Regulated by LPAL2

**Figure 3:**
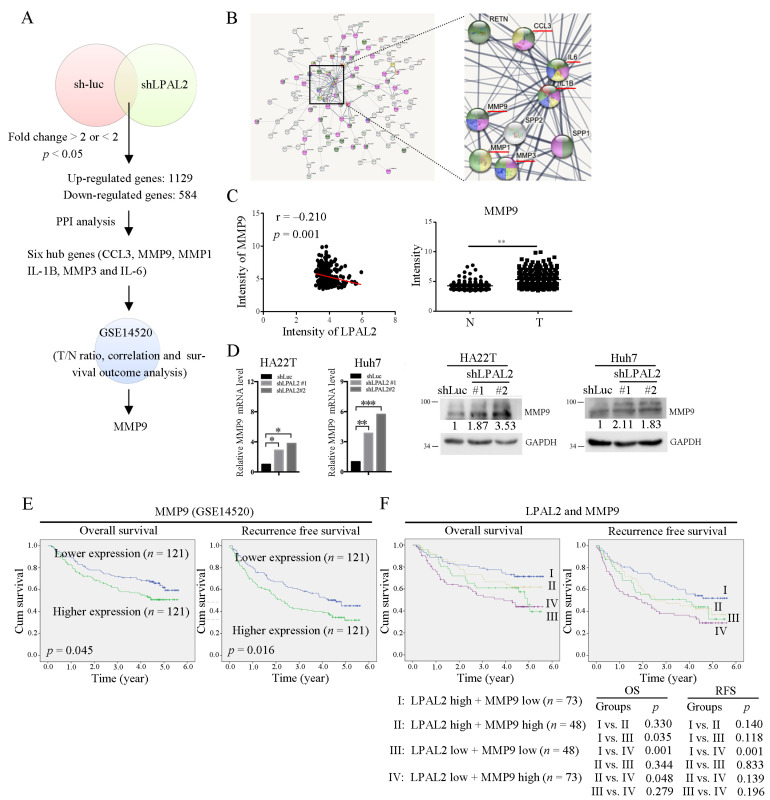
MMP9 is upregulated by LPAL2 depletion. (**A**) LPAL2-regulated genes in LPAL2-knockdown HCC cells, as determined by microarray profiling analysis. Pathway analysis revealed six hub genes, *CCL3*, *MMP9*, *MMP1*, *IL-1B*, *MMP3*, and *IL-6*. The expression levels of these genes and their correlation with LPAL2 in HCC were analyzed in the GSE14520 dataset. (**B**) PPI network of differentially expressed genes in LPAL2-knockdown cell line is shown. (**C**) Left panel: Correlation between LPAL2 and MMP9 in GSE14520, calculated by Pearson correlation analysis. Right panel: Intensity of MMP9 in GSE14520. **, *p* < 0.01. (**D**) MMP9 mRNA (left panel) and protein (right panel) level in LPAL2-knockdown cell lines, as determined by qRT-PCR and Western blot analysis, respectively. *, *p* < 0.05 and ***, *p* < 0.001 (**E**) Kaplan–Meier analysis showing the relationship between MMP9 expression and the overall survival and recurrence-free survival of HCC patients. The median value of MMP9 was used as a cutoff. (**F**) Combined effects of LPAL2 and MMP9 on survival outcomes, determined by Kaplan–Meier analysis using a log-rank test.

To determine how LPAL2 regulates tumor growth, cell migration, invasion and metastasis, we identified LPAL2-regulated target genes through microarray profiling analysis (Figure 3A). In addition, those genes regulated by LPAL2 were subjected to PPI analysis using the STRING bioinformatics tool. Notably, six hub genes, including C-C motif chemokine ligand 3 (*CCL3*), matrix metalloproteinase 9 (*MMP9*), *MMP1*, *MMP3*, interleukin 1β (*IL1B*) and *IL6*, were identified and shown to be enriched by network analysis (Figure 3B). A subsequent Pearson correlation coefficient analysis using the GSE14520 dataset showed that, of these six genes, only MMP9 was significantly negatively correlated with LPAL2 (Figure 3C, left panel). Moreover, MMP9 was highly expressed in HCC tissues compared with adjacent normal tissues (Figure 3C, right panel). Notably, in this context, MMP proteins have been identified as crucial contributing factors to cell migration and invasion [11]. Furthermore, MMP9 mRNA and protein were upregulated in LPAL2-depleted HA22T and Huh7 cell lines (Figure 3D), indicating that the gene encoding MMP9 may be a direct target of LPAL2.

### 3.4. Clinical Correlation of LPAL2 with MMP9

Notably, patients whose tumors showed higher expression of MMP9 exhibited significantly poorer overall survival and recurrence-free survival than those with tumors showing lower expression of MMP9 (Figure 3E). In light of the negative correlation between LPAL2 and MMP9 in HCC tissues (Figure 3B), we further evaluated the combined effects of LPAL2 and MMP9 on clinical outcomes. Using the median value as a cutoff, we classified patients into four groups: Group I, LPAL2-high and MMP9-low (*n* = 73); Group II, LPAL2-high and MMP9-high (*n* = 48); Group III, LPAL2-low and MMP9-low (*n* = 48); and Group IV, LPAL2-low and MMP9-high (*n* = 73)). Patients in Group I showed significantly better overall survival and recurrence-free survival than those in Group IV (Figure 3F). Thus, clinical investigations support the critical pathological role of the LPAL2/MMP9 axis in HCC.

### 3.5. LPAL2 Alleviates Doxorubicin Resistance and Cancer Stem Cell Phenotypes

**Figure 4:**
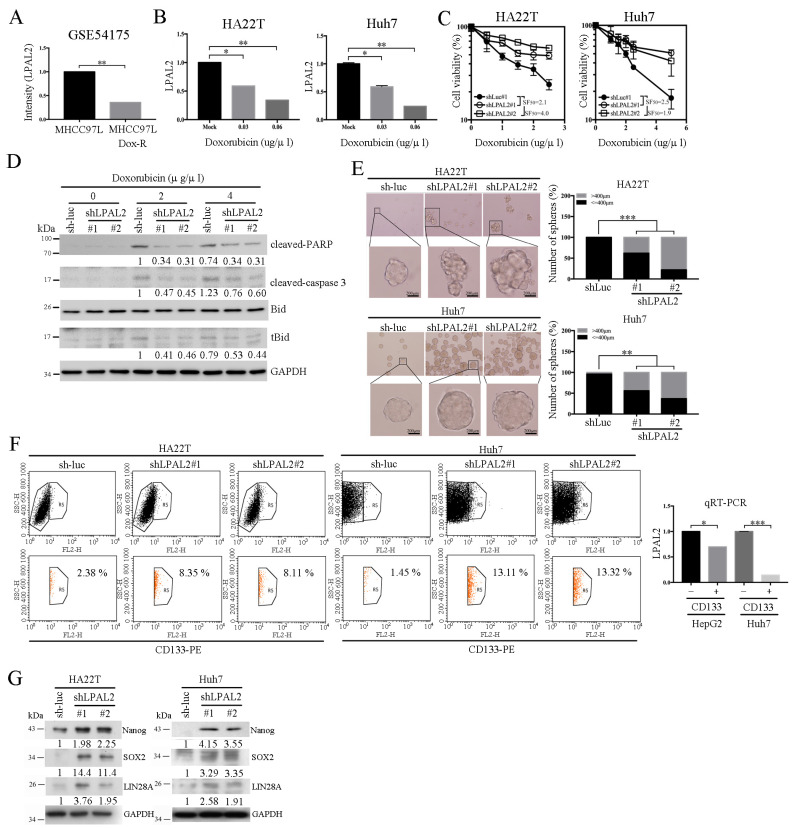
LPAL2 is involved in drug resistance and cancer stem cell phenotypes. (**A**) LPAL2 expression in a doxorubicin-resistant hepatoma cell line, as determined in the online-available GSE54175 dataset. *, *p* < 0.05; **, *p* < 0.01. (**B**) Expression level of LPAL2 in HA22T and Huh7 cells treated with doxorubicin, measured using qRT-PCR. 18s rRNA was used as a loading control. (**C**) Cell viability in LPAL2-knockdown cell lines treated with/without doxorubicin was determined by MTT assay. (**D**) Expression levels of PARP, cleaved-PARP, Bid, truncated Bid (tBid) and caspase 3 proteins in LPAL2-depleted HA22T cells with/without doxorubicin treatment were measured by Western blot analysis. GAPDH was used as a loading control. (**E**) Sphere-formation assay in LPAL2-depleted hepatoma cell lines was determined. Sphere size is calculated and shown. **, *p* < 0.01; ***, *p* < 0.001. (**F**) Upper panel: CD133 expression in LPAL2 knockdown stable cell line was assayed by flow cytometry. Lower panel: LPAL2 expression in CD133-positive and -negative hepatoma cell lines was measured using qRT-PCR. 18s rRNA was used as a loading control. *, *p* < 0.05; ***, *p* < 0.001. (**G**) Expression levels of Nanog, SOX2 and LIN28A proteins in LPAL2-knockdown stable cell lines were measured using Western blot analysis. GAPDH was used as a loading control.

A previous study demonstrated that poorer patient survival outcomes were positively associated with drug-resistant phenotypes [12]. To test whether LPAL2 is involved in drug-resistant phenotypes, we first analyzed LPAL2 expression using an online-available dataset (GSE54175). This analysis showed that LPAL2 expression was decreased in the doxorubicin-resistant hepatoma cell line, MHCC97L-Dox-R (Figure 4A). To verify this, we evaluated LPAL2 expression in hepatoma cell lines treated with or without doxorubicin, and found that LPAL2 expression was repressed by doxorubicin treatment in HA22T and Huh7 cell lines (Figure 4B). Moreover, the knockdown of LPAL2 in HA22T and Huh7 cell lines blocked doxorubicin-induced cell death (Figure 4C). A subsequent determination of the apoptosis-related markers, Bid, PARP and caspase 3 using Western blot analysis showed that truncated Bid (tBid), cleaved-PARP, and cleaved caspase-3 were repressed in LPAL2-knockdown cell lines treated with doxorubicin (Figure 4D). Increasing evidence supports the concept that cancer stem cells are important contributors to drug resistance [13]. To test this possibility, we performed sphere-formation assays using LPAL2-knockdown cell lines. Notably, LPAL2 knockdown accelerated sphere formation (Figure 4E), indicating that LPAL2 is involved in the cancer stem cell phenotype. CD133 in cancers, including HCC, serves as a cancer stem cell marker. Thus, we isolated CD133-negative and -positive HepG2 cell lines by flow cytometry and measured LPAL2 expression in these cell lines using qRT-PCR. Notably, LPAL2 expression was decreased in CD133-positive cells compared with CD133-negative cells (Figure 4F). Consistent with this, flow cytometry analyses showed that the distribution of CD133 was increased in LPAL2-knockdown cell lines (Figure 4F). Their findings suggest that an LPAL2–CD133 reciprocal negative regulatory phenotype controls sphere formation in HCC. Moreover, the cancer stem cell-related markers, Nanog, SOX2 and LIN28A, were induced by the depletion of LPAL2 (Figure 4G). Collectively, these findings reveal that LPAL2 functions as a suppressor that represses stemness through the modulation of cancer stem cell marker expression.

## 4. Discussion

Non-coding genes, including pseudogenes, were previously considered to be “junk” DNA. However, RNA sequencing technology has provided insight into these non-coding genes, demonstrating their unique functions in regulating cell growth, metastasis, drug resistance, and metabolic reprogramming [4]. In this study, we found that the pseudogene-derived lncRNA LPAL2 is clinically relevant in HCC. Functionally, the knockdown of LPAL2 induces tumor growth, sphere formation, migration, and invasion. Our collective results indicate that LPAL2 acts as a tumor-suppressor lncRNA in HCC.

Increasing evidence supports the conclusion that pseudogene-derived lncRNAs are responsible for controlling biological functions [14]. Cui et al. demonstrated that the pseudogene-derived lncRNA WFDC21P is highly expressed in gastric cancer (GC) tissues [15], and showed that the overexpression of WFDC21P induced cell growth and metastasis in GC cell lines through the modulation of the AKT/GSK3β/β-catenin axis. Another report revealed that the expression of the pseudogene-derived lncRNA surfactant associated 1 (SFTA1P) was lower in GC tissues than in adjacent normal tissues [16]. The ectopic expression of SFTA1P was shown to repress the growth, migration and invasion of GC cell lines, as well as positively regulate the expression of the tumor suppressor P53. Huang and co-workers demonstrated that the lncRNA PTTG3P (pituitary tumor-transforming 3, pseudogene) is highly expressed in HCC [17], and that a higher expression of lncRNA PTTG3P is positively associated with tumor TNM stage, tumor size, and poor survival outcomes. This study also showed that the overexpression of lncRNA PTTG3P induces tumor growth and metastasis in vitro and in vivo, through the modulation of PTTG1/PI3K/AKT signaling. Pan et al. demonstrated that the lncRNA PDPK2P is upregulated in HCC tumors compared with normal tissues [18], and further showed that the ectopic expression of lncRNA PDPK2P promotes tumor growth and metastasis via the PDK1/AKT/caspase 3 cascade. In our study, we established a novel role of LPAL2 in regulating tumor growth, metastasis, sphere formation, and drug resistance. These findings suggest that pseudogene-derived lncRNAs, including LPAL2, are not irrelevant molecules as once thought, but instead are biologically important regulatory factors.

MMP9 is a well-known metastatic inducer important in cancer progression [19]. In the current study, we found, using microarray profiling, that the gene encoding MMP9 is a direct target of LPAL2. Validation experiments confirmed that MMP9 mRNA is dramatically induced by the depletion of LPAL2, suggesting that MMP9 is regulated by LPAL2 at the transcriptional level. Notably, LPAL2 levels were significantly negatively correlated with MMP9 levels in HCC specimens. Moreover, an analysis of the combined effects of LPAL2 and MMP9 showed that survival outcomes were better in HCC patients with a higher expression of LPAL2 and lower expression of MMP9 than in other groups. These findings suggest that the LPAL2/MMP9 axis could serve as a novel prognostic marker in HCC patients.

It has been reported that drug resistance and cancer stem cell-related phenotypes are positively associated with poor survival outcomes [20]. Here, we found that knockdown of LPAL2 in hepatoma cells impaired doxorubicin-induced cell death by modulating the expression of apoptosis-related markers. It also facilitated sphere formation through the upregulation of Nanog, SOX2, and LIN28A expression. Notably, LPAL2 expression was decreased in CD133-negative hepatoma cells, suggesting the involvement of LPAL2 in cancer stem cell-related functions. These associations may account for the poor survival outcomes in HCC patients with decreased LPAL2 expression. Doxorubicin treatment has been shown to enhance MMP9 expression in H9c2 rat heart-derived embryonic myocytes [21], an effect that was reported to be mediated by p38 kinase, whereas NF-κB has been reported to promote MMP9 expression and 5′-fluorouracil resistance in colorectal cancer cell lines [22]. These indirect associations suggest that LPAL2 depletion induces MMP9 expression, which, in turn, contributes to the drug-resistant phenotype in HCC cell lines. The direct, transcriptional effects of LPAL2 on MMP9 expression should be fully addressed in future studies.

## 5. Conclusions

In conclusion, our findings advance the novel concept that LPAL2 is a tumor suppressor in HCC. Specifically, we showed that the knockdown of LPAL2 accelerates tumor growth and metastasis, and that MMP9 is directly regulated by LPAL2. Moreover, our clinical findings demonstrated that survival outcomes are improved in HCC patients with a higher expression of LPAL2 and lower expression of MMP9. Taken together, our experimental results and clinical observations support targeting LPAL2 and MMP9 as a therapeutic approach for the treatment of HCCs.

## Figures and Tables

**Table 1:** Clinicopathological correlations of LPAL2 in HCC specimens.

Parameters	*n* = 157	LPAL2 Mean ^a^ ± SE	*p* ^b^
Age (years)			
<65	99	0.7794 ± 0.1007	0.8944
≥65	58	0.6136 ± 0.0790	
Gender			
Male	79	0.5916 ± 0.0754	0.0134
Female	78	0.8462 ± 0.1171	
Cirrhosis			
No	94	0.7073 ± 0.1004	0.2685
Yes	63	0.7344 ± 0.0903	
AFP			
Low	98	0.6554 ± 0.0701	0.5379
High	55	0.8294 ± 0.1552	
Tumor type			
Solitary	125	0.7400 ± 0.0838	0.9809
Multiple	32	0.6329 ± 0.1036	
Tumor size			
<5 cm	91	0.7961 ± 0.1032	0.2172
≥5 cm	66	0.6107 ± 0.0856	
Vascular invasion			
No	91	0.7787 ± 0.1017	0.1937
Yes	66	0.6346 ± 0.0896	
Pathological stage			
I	75	0.7298 ± 0.0859	0.5887
II	51	0.8018 ± 0.1627	
III	31	0.5521 ± 0.1050	
Grading			
1	4	0.7812 ± 0.1094	0.2256
2	110	0.6516 ± 0.0686	
3	43	0.8824 ± 0.1848	

a: Mean of LPAL2 expression (T/N ratio); b: Mann–Whitney U test (for two groups) or Kruskal–Wallis test (for >two groups).

## Data Availability

Online-available datasets, GSE101679, GSE62232, GSE14520 and GSE54175 were used for analyzed.

## Supplementary Materials

The following supporting information can be downloaded at: https://www.mdpi.com/article/10.3390/cells11162610/s1. Table S1: The primers used in this study.
