# Supplementary material for: LPAL2 Suppresses Tumor Growth and Metastasis of Hepatocellular Carcinoma by Modulating MMP9 Expression
Source: Cells. 2022 Aug 22;11(16):2610. doi: 10.3390/cells11162610 (PMC9406458; doi:10.3390/cells11162610)
Supplement: Supplementary file 1 [file cells-11-02610-s001.zip › cells-1815967-supplementary.pdf]

Supplementary Table 1. The primers were used in this study.

Primers for the qRT-PCR
human LPAL2 forward primer: 5'-AGGCTCCTTCCGAACAAGTAAAA-3'<br>human LPAL2 reverse primer: 5'-CGTGCAGCACTAAAGGCTTCT-3'<br>human MMP9 forward primer: 5'-CCCTGGAGACCTGAGAACCA -3’<br>human MMP9 reverse primer: 5'-TTCGACTCTCCACGCATCTCT -3’<br>human 18S rRNA forward primer: 5'-CGAGCCGCCTGGATACC-3'<br>human 18S rRNA reverse primer: 5'-CCTCAGTTCCGAAAACCAACAA-3'
**Primers for shRNA construction**
LPAL2 shRNA#1 forward primer: 5’-CCGGGATACTTCCAAACAAGCACTGCTCGAGCAGTGCTTGTTTGGAAGTATCTTTTT-3’<br>LPAL2 shRNA#1 reverse primer: 5’-AATTAAAAAGATACTTCCAAACAAGCACTGCTCGAGCAGTGCTTGTTTGGAAGTATC-3’<br>LPAL2 shRNA#2 forward primer: 5’-CCGGTGATGCAGAAGCCTTTAGTGCCTCGAGGCACTAAAGGCTTCTGCATCATTTTT-3’<br>LPAL2 shRNA#2 reverse primer: 5’-AATTAAAAATGATGCAGAAGCCTTTAGTGCCTCGAGGCACTAAAGGCTTCTGCATCA-3’
